# Epilepsy and *BRAF* Mutations: Phenotypes, Natural History and Genotype-Phenotype Correlations

**DOI:** 10.3390/genes12091316

**Published:** 2021-08-26

**Authors:** Domenica I. Battaglia, Maria Luigia Gambardella, Stefania Veltri, Ilaria Contaldo, Giovanni Chillemi, Chiara Veredice, Michela Quintiliani, Chiara Leoni, Roberta Onesimo, Tommaso Verdolotti, Francesca Clementina Radio, Diego Martinelli, Marina Trivisano, Nicola Specchio, Charlotte Dravet, Marco Tartaglia, Giuseppe Zampino

**Affiliations:** 1 Child Neurology and Psychiatry Unit, Fondazione Policlinico Universitario Agostino Gemelli, IRCCS, 00168 Rome, Italy; marialuigia.gambardella1@guest.policlinicogemelli.it (M.L.G.); stefaniaveltri@hotmail.it (S.V.); ilaria.contaldo@policlinicogemelli.it (I.C.); chiara.veredice@policlinicogemelli.it (C.V.); charlotte.dravet@free.fr (C.D.); 2 Dipartimento Scienze della Vita, Università Cattolica del Sacro Cuore, 00168 Rome, Italy; michela.quintiliani@yahoo.it (M.Q.); giuseppe.zampino@unicatt.it (G.Z.); 3 Department for Innovation in Biological Agro-Food and Forest Systems (DIBAF), University of Tuscia, 01100 Viterbo, Italy; gchillemi@unitus.it; 4 Institute of Biomembranes, Bioenergetics and Molecular Biotechnologies, Centro Nazionale delle Ricerche, 70126 Bari, Italy; 5 Center for Rare Disease and Congenital Defects, Fondazione Policlinico Universitario Agostino Gemelli, IRCCS, 00168 Rome, Italy; chiara.leoni@policlinicogemelli.it (C.L.); roberta.onesimo@policlinicogemelli.it (R.O.); 6 Department of Radiology Fondazione Policlinico Universitario Agostino Gemelli, IRCCS, 00168 Rome, Italy; tommaso.verdolotti@policlinicogemelli.it; 7 Genetics and Rare Diseases Research Division, Ospedale Pediatrico Bambino Gesù, IRCCS, 00146 Rome, Italy; fclementina.radio@opbg.net (F.C.R.); marco.tartaglia@opbg.net (M.T.); 8 Division of Metabolism, Ospedale Pediatrico Bambino Gesù, IRCCS, 00165 Rome, Italy; diego.martinelli@opbg.net; 9 Department of Neuroscience, Ospedale Pediatrico Bambino Gesù, IRCCS, 00165 Rome, Italy; marina.trivisano@opbg.net (M.T.); nicola.specchio@opbg.net (N.S.)

**Keywords:** cardiofaciocutaneous syndrome, *BRAF*, epilepsy, status epilepticus, hyperekplexia, genotype–phenotype correlations

## Abstract

Objective: Cardiofaciocutaneous syndrome (CFCS) is a rare developmental disorder caused by upregulated signaling through the RAS-mitogen-activated protein kinase (MAPK) pathway, mostly resulting from de novo activating *BRAF* mutations. Children with CFCS are prone to epilepsy, which is a major life-threatening complication. The aim of our study was to define the natural history of epilepsy in this syndrome and exploring genotype–phenotype correlations. Methods: We performed an observational study, including 34 patients with molecularly confirmed diagnosis (11 males, mean age: 15.8 years). The mean follow-up period was 9.2 years. For all patients, we performed neurological examination, cognitive assessment when possible, neuroimaging, electrophysiological assessment and systematic assessment of epilepsy features. Correlation analyses were performed, taking into account gender, age of seizure onset, EEG features, degree of cognitive deficits, type of mutation, presence of non-epileptic paroxysmal events and neuroimaging features. Results: Epilepsy was documented in 64% of cases, a higher prevalence compared to previous reports. Patients were classified into three groups based on their electroclinical features, long-term outcome and response to therapy. A genotype–phenotype correlation linking the presence/severity of epilepsy to the nature of the structural/functional consequences of mutations was observed, providing a stratification based on genotype to improve the clinical management of these patients.

## 1. Introduction

The RAS/mitogen-activated protein kinase (MAPK) pathway controls a wide array of cellular processes, and its proper function is required for the accomplishment of developmental programs as well as for neuronal function [1,2]. Germline mutations in components of the RAS/MAPK cascade cause a family of clinically related developmental disorders, collectively known as “RASopathies” [3,4,5]. These multi-system diseases are characterized by variable neurological features, including developmental delay (DD)/intellectual disability (ID) and behavioral anomalies. Children affected by one of these disorders, cardiofaciocutaneous syndrome (CFCS, OMIM #PS115150), show pervasive neurological involvement, with hypotonia and moderate/severe DD/ID as major features [6]. In these children, epilepsy is a clinically relevant complication, being reported in a variable proportion of affected individuals [6,7,8,9]. In the majority of cases, CFCS is caused by de novo activating mutations in *BRAF* (MIM *164757), encoding a serine/threonine protein kinase functioning as a RAS effector [10,11]. Seizures have been estimated to occur in between 15–50% of patients with *BRAF* mutations [6,7,8,9]. Many patients require multidrug therapy and show drug resistance, indicating that difficult seizure control is a clinically relevant complication of the disorder. Moreover, a subset of these patients is affected by developmental and epileptic encephalopathy [12,13,14].

Due to the difficulty in obtaining large and unselected cohorts, a systematic assessment of the electroclinical features, natural history of disease, long-term outcome and response to therapy is lacking. Similarly, no information on possible genotype–phenotype correlations to stratify risk of these patients is currently available. Here, we provide a systematic assessment of the epileptic profile and natural history of disease in a large, unselected and clinically well-characterized cohort of patients with *BRAF*-related CFCS, and propose a genotype-based stratification of patients to improve clinical management and care.

## 2. Materials and Methods

### 2.1. Study Population

The observational longitudinal study was performed on 34 patients with molecularly confirmed clinical diagnosis of CFCS [15]. The detailed characterization of the study cohort is provided as Supplemental Information.

### 2.2. Study Design

A team composed of three pediatric neurologists examined all neurological and electroclinical records, and contributed to the developmental assessment. An experienced radiologist scanned the neuroimaging records.

The clinical study included an assessment of the epilepsy features, taking into account the age of onset, semiology and frequency of seizures, recurrence of epileptic status and response to treatment, following the ILAE criteria [16,17,18]. We considered status epilepticus (SE) as refractory when continuous or repetitive seizures did not respond to first- and second-line anticonvulsant drugs [19].

The electrophysiological assessment consisted of video-polygraphy recording performed with 21 EEG electrodes according to the 10/20 International system, ECG and deltoid surface EMG channels. All patients were assessed by at least one awake and sleep recording lasting more than 1 h; 16 patients underwent one or more 24 h video-EEG monitoring. In 14 patients, ictal video-EEG recordings were also obtained. Brain MRI was performed at least once in 28 patients.

### 2.3. Structural Analysis

The location of the residues mutated in the study cohort was assessed using the three-dimensional structure recently solved by cryo-electron microscopy of the inactive BRAF/MEK1/14-3-3 complex (PDB 6nyb) [20,21], which was visualized using the VMD software (http://www.ks.uiuc.edu/Research/vmd/).

### 2.4. Statistical Analysis

Correlation analyses were performed taking into account gender, age at seizure onset, EEG features (normal vs. abnormal background activity; absence vs. presence of focal, multifocal or diffuse paroxysmal activities), ID (mild, moderate, severe, profound), type of mutation, absence vs. presence of non-epileptic paroxysmal events (PNE), such as blinks, axial myoclonic and/or exaggerated startle responses, neuroimaging features (normal, cortical atrophy associated or not to hippocampal sclerosis or other abnormalities) and distribution of mutations among phenotypic classes.

Chi square test (χ^2^) was employed for nominal variables. Fisher’s exact test was used when the prerequisites for χ^2^ test couldn’t be met. Student’s *t*-test was employed for continuous variables. A *p*-value less than 0.05 was considered statistically significant.

## 3. Results

The relative prevalence of each *BRAF* mutation in the studied cohort reflected the distribution reported in the NSEuroNet database (https://nseuronet.com/php/), an international repository dedicated to RASopathies, indicating that the composition of the subjects enrolled in the study is representative of the general CFCS population with *BRAF* mutations. Six patients (18.0%) were heterozygous for the most common CFCS-associated variant, p.Lys257Gln (24.0% in the NSEuroNet database), while the other recurrent amino acid substitutions observed in the present cohort (p.Trp531Cys, p.Phe595Leu, p.Lys601Gln and p.Asp638Glu) had consistently been reported in multiple cases in the NSEuroNet database. Similarly, the relative prevalence of mutations affecting residues located in the two major functional domains of the kinase (i.e., the cysteine-rich domain [CRD] and conserved region 3 [CR3], see below), which represent the mutational hot spots of CFCS-causing *BRAF* mutations, was comparable (CRD, 32.4% vs. 37.7%; CR3, 67.7% vs. 62.0%).

### 3.1. Neurological Data

The collected neurological features are reported in Table 1. All subjects presented DD, with twenty of them being able to either walk autonomously or being able to walk when assisted and three having language ability. Two patients who had reached the ability to walk showed severe regression of the acquired motor skills after recurrent epileptic status. Global hypotonia was observed in eight cases, tetraparesis in nineteen and ataxia in five. Fourteen patients had eye movement disorders (strabismus in 13 patients; nystagmus in two patients). Cognitive impairment was profound in eight subjects, severe in twelve, moderate in seven and mild in five individuals. Seven patients showed autistic traits. Twenty patients had sleep disorders characterized by difficulty to fall asleep and frequent nocturnal awakening. Non-epileptic paroxysmal events, consisting of an exaggerated startle response to visual, auditory and somesthetic stimuli (hyperekplexia), were documented in 15 subjects. 

### 3.2. Electroclinical Data

Epilepsy was observed in 22 patients (64.7%). Among them, 10 subjects (mean age at follow up 11.5 years ± 6.2, range 3–23 years, 6 males) presented seizure onset before 4 years of age (mean age 12.3 months, range from one day to four years), multiple seizure types, high seizure frequency (>1/week), recurrent SE, and seizures not responding to combined multidrug therapy. Twelve patients (mean age at follow up 15.6 years ± 6.7, range 6–27 years, 2 males) presented with sporadic (<1/month) seizures with age at onset from 2 years to 18 years (mean age 9 years), absence of epileptic status after antiepileptic treatment in their clinical history and good response to antiepileptic drugs. Finally, twelve patients (mean age at follow up 21.1 years ± 14.9, range 2–53 years, 3 males) did not present epilepsy until last assessment.

Based on the collected epileptic features (age at onset, semiology and frequency of seizures, recurrence of epileptic status and resistance/response to treatment), we classified the epileptic phenotypes into three groups (Table 1): group 1 (Pts 1–10), including subjects with severe epileptic phenotype; group 2 (Pts 11–22), comprising patients with mild epileptic phenotype; group 3 (Pts 23–33), consisting of patients older than six years without epilepsy, with/without EEG abnormalities (epilepsy-free group). While Pt34 was epilepsy-free at last assessment, the patient was not included in group 3 because of her young age. Electroclinical data are shown in Table S1.

#### 3.2.1. Clinical Epilepsy Data

Group 1 (severe epileptic phenotype): Mean follow-up duration was 10.9 years ± 6.2 (range: 33 months to 22 years). Seizure onset occurred within the first 2 years of life in nine subjects, and at 4 years in the remaining patient. After seizure onset, a long seizure-free interval (mean duration of 5.2 years, ranging from 8 months to 7 years) was observed in eight patients. After this interval, high-frequency (daily or weekly) seizures occurred in all patients. Semiology at onset consisted of focal seizures (seven patients) and epileptic spasms (two patients). During follow-up, all patients presented multiple seizure types. Four different types of seizures were observed: (1) focal motor onset seizures with different semiology (all patients), the most peculiar represented by hyperkinetic seizures, starting with massive myoclonia, startle-like, followed by axial and limb tonic component, finalistic and afinalistic limb movements, blinking and oral automatisms. Duration was variable, lasting 10–30 s, sometimes repeatedly occurring in clusters up to 15–30 min; (2) generalized myoclonic seizures (six patients); (3) epileptic spasms (four patients); (4) generalized tonic seizures (one patient). All subjects presented with recurrent SE, followed by developmental regression in four patients (not appreciable in the remaining six patients due to the profound ID preceding epilepsy onset). SE was focal motor in all cases, associated to convulsive SE (focal onset evolving to bilateral convulsive SE) in three of them. Refractory SE occurred in four patients, evolving in exitus in two subjects (Pt7 and Pt9). The seizures were either spontaneous or triggered by fever, cold, urination or sensorial stimulations (e.g., tactile or auditory). According to the ILAE criteria, epilepsy types were classified as combined focal and generalized in seven cases and focal in three.

At outcome, in all patients, weekly or daily seizures persisted despite polytherapy with antiepileptic drugs (AEDs), steroids, ketogenic diet and vagus nerve stimulation (VNS) (one case).

Group 2 (mild epileptic phenotype): Mean follow-up duration was 9.7 years ± 7 (range five years to 25 years). Onset of seizures occurred between 6 and 10 years in five patients, and between 11 and 18 years in four patients. Only three patients presented seizure onset during the first 4 years of life. Seizures at onset consisted of focal onset seizures (ten patients) and generalized myoclonic seizures (two patients). During the follow-up, only one seizure type was observed in nine patients (focal onset seizures in eight, and massive myoclonia in one), and two seizure types (focal onset seizures and massive myoclonia) in the remaining three subjects. One isolated focal epileptic status was observed in two subjects before starting antiepileptic therapy, with or without prominent motor symptoms (Pt20 and Pt19, respectively). Similarly to group 1, seizures were either spontaneous or triggered by fever and various sensorial stimulations. Epilepsy types were classified as focal in eight patients, combined focal and generalized in three, and generalized in one. All subjects were seizure-free at follow up; nine patients had been treated with monotherapy, one with bitherapy (Pt19) and two had not been treated (Pt14 and Pt22).

Group 3 (epilepsy-free): Mean follow-up duration was 8 years ± 4.3 (range 2 years to 17 years). No epileptic seizures had been reported during follow-up.

#### 3.2.2. Electrophysiological Assessment

Interictal EEG: In group 1, at seizure onset, EEG was available in five patients. Background activity was normal for age in two (Pt4 and Pt6) and slow in one (Pt1), hypsarrhythmic pattern was observed in two (Pt3 and Pt7) and temporal spikes in one case (Pt1). During follow-up, all patients but one showed slow and poor organized, sometimes asymmetric background activity, with progressive disappearance of physiological sleep pattern. Bursts of focal high amplitude fast activity during wakefulness and sleep were observed in five patients. All patients showed an increase of epileptiform abnormalities, consisting of irregular and high voltage spike, spike-wave and polyspike-wave discharges. They were focal, multifocal or clustering in symmetric or asymmetric diffuse discharges (Figure S1). Four patients also showed diffuse discharges of high amplitude slow waves with intermingled irregular spike-waves and polyspike-waves. The paroxysmal abnormalities were activated by slow sleep in all patients; in three patients, a paroxysmal alternating pattern was observed, which was characterized by diffuse irregular arrhythmic spike-wave and poly spike-wave discharges, lasting 1–3 s and alternating with brief and low voltage activity.

In group 2, the EEG showed a better organized background activity with a reactive posterior alpha rhythm. In four subjects, unusual activities characterized by fast activity or theta rhythmic activity on anterior regions and giant spindles during NREM sleep were observed. Paroxysmal abnormalities were generally observed during sleep and were characterized by focal spikes and sharp waves in eight subjects, multifocal spikes in one patient and diffuse spike and spike-wave discharges and combined focal and diffuse spike-wave discharges two subjects (Figure S2).

In group 3, all subjects showed normal background activity. Paroxysmal activity was observed in two subjects, consisting of isolated spikes and sharp waves in the posterior regions (Pt32 and Pt33).

**Figure 1:**
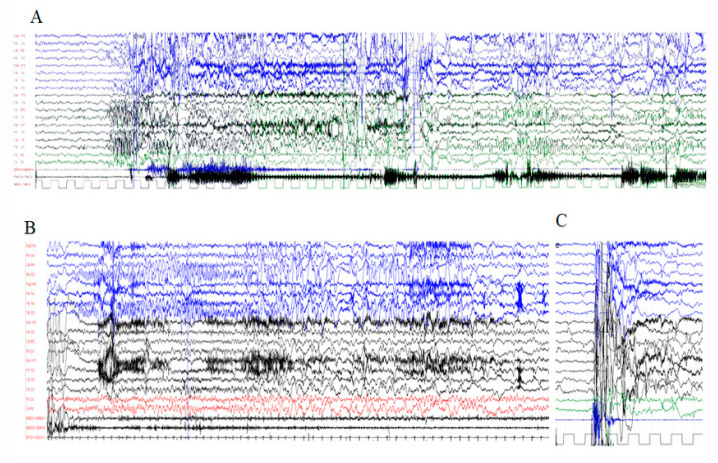
Group1: Ictal EEG in a 6-year-old boy (Pt5, Lys601Gln). (**A**) Left hyperkinetic seizure: Ictal discharge characterized by rapid spikes, starting on the left middle and posterior areas and spreading to the same right regions, followed by diffuse slow waves. After 10 s, a discharge of rapid SW started on the left occipital regions, lasting 20 s. (**B**) Right focal seizure characterized by sustained right deviation of the eyes and head, followed by eye clonic movements. Ictal discharge, starting in the right posterior regions, characterized by rapid spikes, then by SW and then again by rapid spikes. (**C**) Muscle artifacts associated with a startle response without paroxysmal abnormalities.

**Figure 2:**
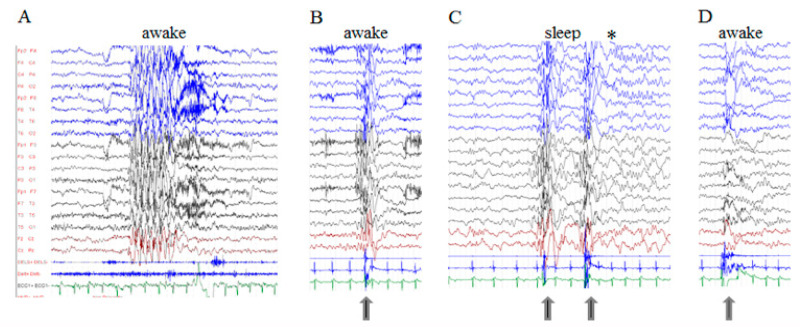
Group 2: Ictal EEG in a 9-year-old girl (Pt16, Trp531Cys). (**A**) Subclinical discharge of diffuse and irregular SW. (**B**) Bursts of diffuse polyspike and polyspike-waves, with anticipated onset over the frontal or posterior areas, accompanied by myoclonic jerks of both arms (arrows) while awake. (**C**) The same polyspike and polyspike-waves recorded during sleep. Note the spindles on the fronto-central regions (*). (**D**) Muscle artifacts associated to a startle response without paroxysmal abnormalities.

Ictal EEG: Ictal EEG were recorded in all patients of group 1 and in six patients of group 2 (Table S1). Focal seizures were recorded in eleven cases (eight within group 1 and three within group 2), with variable onset, either in the frontal or temporal or occipital areas (Figure 1). The hyperkinetic seizures, which were recorded in five group 1 patients, were associated with an ictal discharge starting in one localized area of one hemisphere which then spread to the entire hemisphere or another area of the same or opposite hemisphere (Figure 1). The ways of propagation were variable from one seizure to another in the same patient, even in the same recording. Myoclonic seizures, recorded in ten patients (six in group 1, four in group 2), were associated with irregular and diffuse spike-waves (Figure 2). Epileptic spasms, occurring in clusters, were recorded in four group 1 patients and were usually associated to focal or diffuse slow waves, followed by decremental activity, with superimposed fast activity.

### 3.3. Other Paroxysmal Phenomena

Fifteen out of twenty-two patients with epilepsy had PNE characterized by blinks, axial myoclonia and/or exaggerated startle responses (hyperekplexia). No patient without epilepsy had PNE (Table 1). PNE were spontaneous or triggered by noises and other sensorial stimulations (visual and somesthetic, the most important reactivity being tactile stimulations). As stated by the parents, the beginning of these phenomena occurred in the first year of life. Generally, they were more frequent and complex in patients with very severe epilepsy. Hyperekplexia and epileptic focal hyperkinetic seizures often clinically overlapped, presenting themselves as “startle-like” episodes. During the same video-EEG recording, they could be distinguished only by their EEG-associated features (Figure 1C and Figure 2D). Finally, a reaction to sensorial stimulation characterized by tremor, agitation, screams and autonomic manifestations, with flushing, profuse sweating and tachycardia, was documented in two subjects (Pt4 and Pt21).

### 3.4. MRI Data

**Figure 3:**
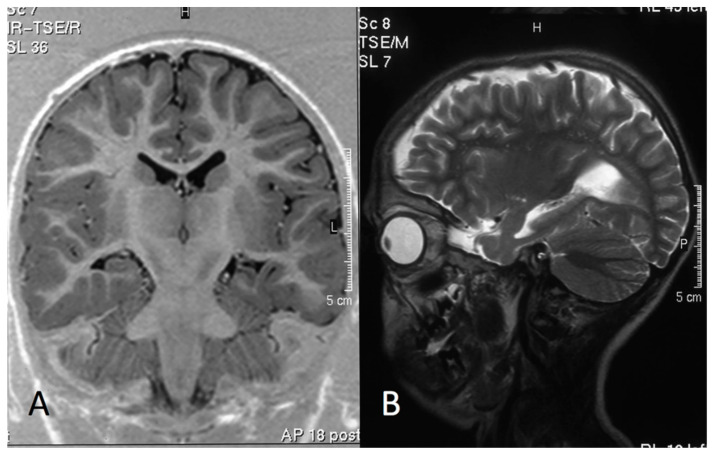
Group 1: MRI of a 11-year-old boy (Pt1, Lys 601Gln). (**A**) Inversion Recovery (IR) Turbo Spin Echo (TSE) Image, coronal plane. (**B**) TSE T2 weighted image, sagittal plane. The MRI shows a small left hippocampus and enlarged temporal horn and collateral sulcus, consistent with left-sided hippocampal sclerosis. Note the mild widening of left sylvian fissure and of the left fronto-temporal cerebrospinal fluid (CSF) spaces, consistent with cortical atrophy.

In twenty-eight patients, brain MRI was performed (Table 1). In group 1, MRI showed cortical atrophy in all patients. In six of them, atrophy was associated to unilateral (Pt1, Pt3, Pt6 and Pt7) (Figure 3) or bilateral (Pt2 and Pt10) hippocampal sclerosis, while in two subjects (Pt7 and Pt8), it was associated to lobe temporal hypoplasia. Pt3 has been submitted to right temporal lobe resection for dysplasia. In group 2, MRI was normal in four subjects. The abnormalities detected in the other eight individuals consisted of hippocampal sclerosis in only one patient who presented with repetitive seizures (Pt19), cortical atrophy in three, cerebellar astrocytoma in one, thin corpus callosum in one, ventriculomegaly in one and periventricular T2-hyperintensity in three (nonspecific signs of gliosis). In group 3, brain MRI was performed in six patients: no abnormalities were observed in two subjects, and minor abnormalities were reported in four subjects, which consisted of thin corpus callosum in three, among which one was associated to cerebellar malrotation, and gliosis in the remaining subject (Table 1).

### 3.5. Structural Analyses and Genotype–Phenotype Correlations

**Figure 4:**
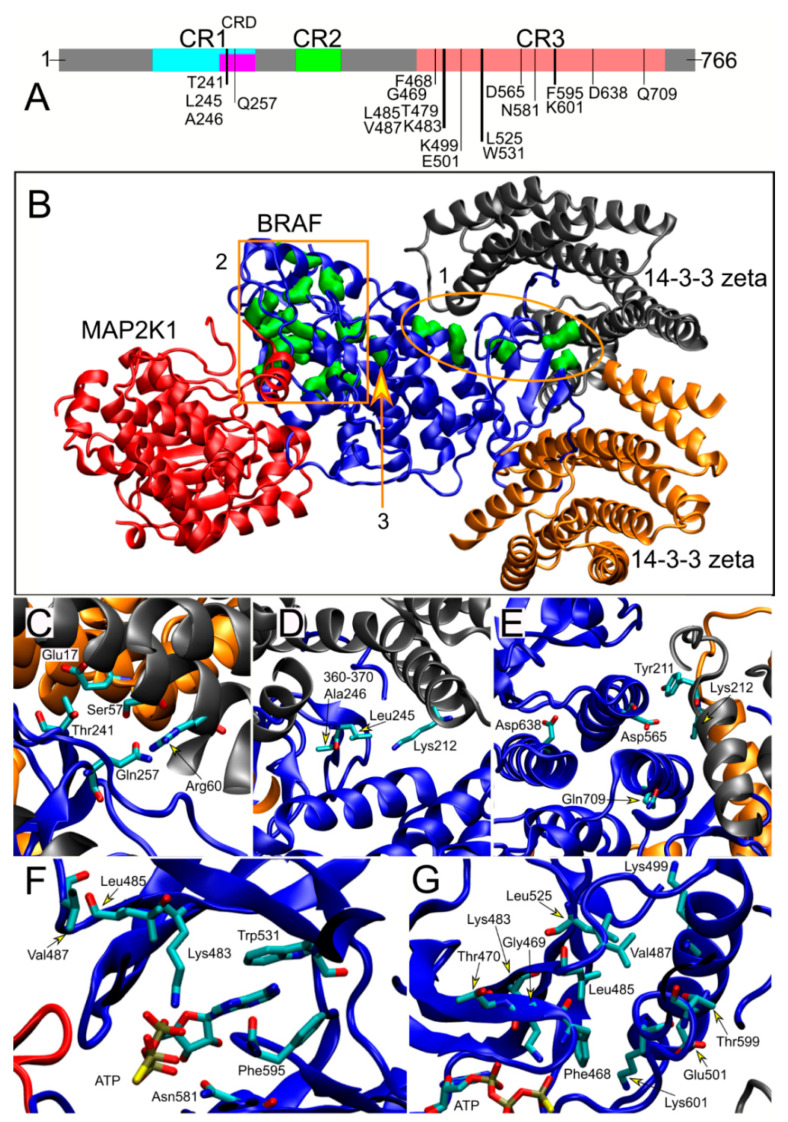
Domain organization of BRAF and location of residues mutated in CFCS. (**A**) Cartoon showing the domain organization of BRAF. The three conserved regions, CR1, CR2 and CR3, are highlighted in cyan, green and orange, respectively. The cysteine-rich domain (CRD, pink), located within CR1, contacts both subunits of the 14-3-3 zeta dimer in the inactive BRAF/MEK1/14-3-3 complex. Binding of the 14-3-3 dimer to phosphorylated Ser365 (CR2) and Ser729 (CR3) maintains BRAF in an autoinhibited state through an intramolecular interaction occurring between CR1 and the kinase domain CR3. In the active state, dephosphorylation of Ser365 results in an “open” conformation of the kinase, which stabilizes its binding with activated RAS proteins. In this activated con formation, a 14-3-3 dimer rearranges to bridge pSer729 of two BRAF proteins, thus driving the formation of the active BRAF dimer (21). The autoinhibited state is stabilized by interaction of residues 598–602 in the activation segment (the so-called ‘inhibitory turn’) with hydrophobic residues in the “GxGxxG” consensus sequence within the glycine-rich loop (residues 464–469) [21]. (**B**) Three-dimensional structure of the BRAF/MEK1/14-3-3 complex from cryo-electron microscopy in its autoinhibited conformation (PDB 6nyb). BRAF, MEK1 and the two monomers of 14-3-3 zeta are highlighted in blue, red, gray and yellow, respectively. The positions of the affected residues are highlighted in green. Two major mutation clusters are apparent: class I (1, ellipse), including residues located in/close to the CRD contacting the 14-3-3 protein (i.e., Thr241, Leu245, Ala246, Gln257, Asp565, Gln709); class II (2, rectangle) including residues mapped in the inhibitory turn region (i.e., Phe468, Gly469, Thr470, Lys483, Leu485, Val487, Lys499, Glu501, Leu525, Trp531, Asn581, Phe595, Thr599, Lys601). The arrow indicates residue Asp638, which is located in the protein core and has been assigned to class III (3). (**C**) In the complex, Thr241 is closer than 5 Å to Ser57 in 14-3-3 zeta monomer 1 and Glu17 in monomer 2. Gln257 is close to Arg60 in 14-3-3 zeta monomer 1. (**D**) Leu245 is close to Lys212 in 14-3-3 zeta monomer 1. The 3D position of residues 360–370 is also indicated. Residues 282–359 and 371–448 are not resolved. (**E**) Lys212 in 14-3-3 zeta monomer 1 is also close to Asp565. Gln709 does not have any 14-3-3 zeta residues closer than 5 Å in this experimental structure, but its substitution to arginine likely alters the interaction with the molecular partner. The 3D position of Asp638 is also shown. This variant is nearly equidistant from CRD and the inhibitory turn region. (**F**,**G**) BRAF variants in the active site region. Note that Phe595 is part of the DFG motif (594–596) in the inhibitory turn region, which plays an essential role in the correct positioning of ATP also in the active open conformation of BRAF. Thr599 and Lys601 are part of the TVKS motif; residues 469–470 are part of the p-loop, responsible for BRAF autophosphorylation.

The BRAF kinase is characterized by three conserved regions, which are shared by the three members of the Raf kinase family (i.e., CR1, CR2 and CR3) (Figure 4A). The N-terminal CR1 domain contains the region mediating binding with activated RAS (RAS-binding domain, RDB) and the cysteine-rich domain (CRD), a motif with regulatory function. The C-terminal CR3 region encompasses the catalytic site and Ser729, which is a binding site for 14-3-3 proteins when phosphorylated. The CR2 region contains a second 14-3-3 binding site (Ser365), which has a key role in regulating the switch between the catalytically inactive and active conformations (Figure 4A) [21].

The inactive and active BRAF/14-3-3 complexes have recently been solved by cryo-electron microscopy) [20], documenting that activating CFCS-causing *BRAF* mutations fall in two main groups: a first cluster affects residues located at the interface of the CR2 region (pSer365) that binds to the regulatory 14-3-3 protein (class I, see ellipse 1 in Figure 4B–E); a second larger cluster perturbs the autoinhibited BRAF conformation affecting residues that are located in the inhibitory turn region (class II, see rectangle 2 in Figure 4B,F–G) [21]. Asp638, which was assigned to a different class (class III, 3 in Figure 4B,E), is located separately. Remarkably, a statistically significant non-random distribution of mutations among the three groups of patients was observed (Fisher’s exact test, *p* = 0.029). Among the thirteen cases sharing class I mutations (CR2 region interacting with the regulatory 14-3-3 protein), we observed a prevalence of patients belonging to the groups 2 and 3 (5 and 7, respectively), with only one mutation (p.Asp565Glu) identified in a single patient from group 1. Conversely, only a small proportion of patients heterozygous for a class II mutation (i.e., affecting residues around the inhibitory turn region) was classified within group 3 (5/18), while the majority consisted of patients with expression of the disease (i.e., groups 1 and 2) (13/18). Even within the latter class of mutations, a strict correlation between severity of symptoms and type of mutations was appreciated, since the two cases with the mutation affecting the key residue within the inhibitory loop, Lys601, showed a severe phenotype. A consistent association of Asp638Glu (class III) with a severe phenotype was also observed, further supporting the occurrence of genotype–phenotype correlations. Overall, these analyses support the idea that mutations that destabilize the autoinhibited state are preferentially associated to severe clinical outcomes, while those affecting the 14-3-3 binding region of the kinase are more frequently associated with absence or a less severe form of epilepsy.

## 4. Discussion

Epilepsy represents a major clinical issue in CFCS, a developmental disorder caused by activating de novo BRAF mutations. Epilepsy occurs in a significant proportion of these patients, with many individuals requiring multidrug therapy and a subset being prone to drug resistant disease and neurodevelopmental regression. While risk-stratified care management of these patients would be advisable, knowledge on allowing the stratification of these patients is still missing. Here, we present a systematic assessment of epilepsy features and the long-term outcome of these patients, and the first evidence of genotype–phenotype correlations, outlining a possible stratification based on the genotype to improve clinical management and care.

In the present series, the percentage of patients with epilepsy was higher compared to what had previously been reported (64% vs. 16–50%) [6,7,8,9,22]. The relative prevalence of mutations in the cohort was in line with the distribution of *BRAF* mutations in the general CFCS population, supporting the unbiased patient enrolment. Rather, we suggest that the higher prevalence of epilepsy is likely explained by the larger size and longer follow-up of the present cohort. Consistently, we observed onset of seizures during late childhood and adolescence in a significant proportion of patients, which is a relevant finding considering a mean age at follow-up lower than 6 years in comparable series.

Our long-term electroclinical study showed a severity gradient of epilepsy according to the age of onset, seizure recurrence, semiology complexity, EEG features and response to AEDs, allowing to identify three patient groups based on the differential expression of the disease. In this classification, group 1 (29% of patients) is characterized by a severe epileptic condition, early-onset of seizures, high seizure frequency, recurrent and refractory SE, slow EEG background activity and usually multifocal and diffuse paroxysmal abnormalities and drug resistance. In this group, seizure types were polymorphic, including focal onset seizures, epileptic spasms and tonic and myoclonic seizures. A particular type of focal onset seizure was documented, consisting of hyperkinetic seizures correlated with migrating discharges, which had not previously been reported in CFCS associated with *BRAF* mutations. Group 2 (35% of patients) presents with a less severe disease characterized by late-onset seizures (late childhood/adolescence), with monomorphic semiology and sporadic recurrence. In this group, epileptic status rarely occurred before AED treatment, and normal EEG background activity and usually drug-responsiveness were observed. Focal epilepsy was recorded in most patients, with combined focal and generalized epilepsy in three cases and generalized epilepsy in one. Finally, group 3 (35% of the present cohort) identifies patients with a normal EEG pattern or minor EEG abnormalities. In the present study, these patients did not present with seizures during their entire follow-up.

As expected, the degree of cognitive and neurological impairment was generally more severe in patients of group 1. Furthermore, the severe epileptic phenotype was associated to a worse neurodevelopmental outcome, including regression in one-third of cases. In group 1, the neurodevelopmental impairment before seizure onset and severe structural brain abnormalities suggest that epileptic activity is not primarily responsible for the DD. Therefore, both epilepsy and encephalopathy appear to be symptoms of the underlying genetic defect. However, the neurodevelopmental regression observed after recurrent epileptic status or high seizure frequency confirmed in one-third of patients denotes the contribution of epileptic activity to the progression of disease. Therefore, referring to the current ILAE definition, the condition observed in group 1 could be defined as developmental and epileptic encephalopathy (DEE), where genetic and epileptic factors play a role in determining developmental impairment [16,17]. In particular, the clinical course of group 1 patients highlights the variable contribution of the “developmental” and “epileptic” component to the “BRAF-related encephalopathy”. These considerations are very relevant to expectations from seizure treatment [23].

Almost half of the patients of the present cohort presented with PNE, which has previously been reported in CFCS [24]. This symptom, which was exclusively observed in patients with epileptic seizures, had a frequency and complexity that correlated with the severity of epilepsy and could be either spontaneous or triggered by sensorial stimulation. Of note, in group 1 patients, overlapping semiology of epileptic and non-epileptic events was documented, indicating the requirement of video-EEG for differential diagnosis.

We noted that six patients with severe epilepsy (group 1) showed unilateral/bilateral hippocampal sclerosis. These findings are suggestive of focal cortical dysplasia (FCD), particularly type IIIa [25]. In Pt3, the histopathology of the resected temporal lobe was compatible with FCD. Of note, BRAF is highly expressed in the hippocampal cortex [26], and pharmacological down-modulation of MAPK signaling inhibits most forms of synaptic plasticity [27], with conditional ablation in forebrain excitatory neurons affecting hippocampus-dependent learning [28]. Oncogenic *BRAF* lesions (i.e., gene fusions and the recurrent Val600Glu amino acid substitution) have also been reported in low grade epilepsy-associated tumors (LEAT) and in FCD [29,30,31,32]. These considerations and the observation of cortical malformations, including pachygyria, heterotopia and cortical temporal dysgenesis in CFCS and other RASopathies [12,33,34,35], further support a possible direct contribution of *BRAF* mutations in altering morphogenetic programs of the brain, which may contribute, in part, to epilepsy in CFCS. This aspect requires further dedicated histopathological studies and assessment in larger cohorts.

A major finding of the present study is the identification of a clinically relevant correlation between the location and type of mutations and severity of disease, which was previously suggested by anecdotical reports and small series [12,24]. Structural assessment of CFCS-causing *BRAF* mutations allowed us to identify two major clusters corresponding to the region of the kinase mediating binding to the regulatory 14-3-3 protein (class I mutation) and the inhibitory turn region (class II mutations). Our data indicate that mutations affecting the 14-3-3 binding region of the kinase are more frequently associated with absence of epilepsy or an overall less severe phenotype, while those destabilizing the autoinhibited state of the active site are preferentially associated with a more severe epileptic outcome. A data review from the literature was performed to validate the observed correlation. Overall, 21 subjects with detailed clinical and molecular characterization, and age > 6 years without seizures, could be considered (Table S2). Classification of these subjects into the three here-defined groups, based on their clinical presentation, allowed to confirm the significant enrichment of class II mutations in patients with severe epileptic phenotype (group 1), and a higher prevalence of class I mutations among patients with less severe epileptic phenotype (group 2) or without epilepsy (group 3) (Fisher’s exact test, *p* = 0.003), confirming the correlation identified in the present series. This genotype–phenotype correlation provides a risk management tool to stratify patients. Prompt pre-symptomatic identification of patients with class II *BRAF* mutations (i.e., higher risk of developing epilepsy or expressing a severe epileptic phenotype) is expected to help clinicians to a more effective disease management in terms of diagnosis, treatment and care, reducing the risks of associated complications and improving health outcomes.

## 5. Conclusions

Our data indicate that epilepsy occurs in CFCS more commonly than previously reported, and that patients can be classified into different groups based on their electroclinical features, long-term outcome and response to therapy. The neurocognitive impairment and regression occurring in patients with severe epilepsy may be related to cortical dysplasia and hippocampal sclerosis, which are likely genetically determined and in turn play a role in the epileptogenic process. Finally, a genotype–phenotype correlation linking the presence/severity of epilepsy to the nature of the structural/functional consequences of mutations was observed, providing a stratification based on genotype to improve clinical management of these patients.

## Figures and Tables

**Table 1:** Demographic, genetic and clinical characterization of the study cohort.

Patient (Age; Sex)	Follow-Up (y)	BRAF Amino Acid Substitution	Mutation Class	Age of Seizure Onset	Epilepsy Type	Neurological Features	ID/Behavioral Disorders	PNE	SD	Brain MRI
**Group 1**										
#1 (23 y; M ^†^)	23	Lys601Gnl	II	2 m	Combined focal and generalized	Tetraparesis, nystagmus	Profound Regression	Hpx	Yes	CA, HS
# 2 (17 y; M)	15	Asp638Glu	III	2 y	Combined focal and generalized	Tetraparesis, dystonia, nystagmus	Profound/AT	Hpx	Yes	CA, bilateral HS, gliosis
# 3 (15 y; M)	15	Asp638Glu	III	5 m	Combined focal and generalized	Tetraparesis	Profound/AT	Hpx	Yes	CA, HS, cortical dysplasia
#4 (14 y; F)	12	Phe595Leu	II	1.5 y	Focal	Tetraparesis	Profound/AT	Hpx	Yes	CA, gliosis
#5 (13 y; M)	11	Lys 601Gnl	II	1.5 y	Combined focal and generalized	Tetraparesis, strabismus	Severe	Hpx	Yes	CA
#6 (12 y; F)	7	Asp638Glu	III	1.5 y	Combined focal and generalized	Tetraparesis, strabismus	Severe Regression	Hpx	Yes	CA, HS
#7 (7 y; F ^†^)	6	Phe595Leu	II	7 m	Combined focal and generalized	Tetraparesis, strabismus	Profound Regression	Hpx	Yes	CA, HS, hippocampal hypoplasia
#8 (6 y; F)	6	Val487Gly	II	8 day	Focal	Tetraparesis	Profound/AT	Hpx	Yes	CA, hippocampal hypoplasia
#9 (5 y; M ^†^)	NA	Asp565Glu	I	4 y	Combined focal and generalized	Tetraparesis, strabismus	Profound	Hpx	NA	CA, thin CC
#10 (3 y; M)	3	Pro468Ser	II	1 m	Focal	Tetraparesis	Profound/AT	NA	Yes	CA + bilateral HS
**Group 2**										
#11 (27 y; F)	25	Trp531Cys	II	2 y	Focal	Ataxia, strabismus	Severe	NA	NA	Normal
#12 (23 y; M)	10	Thr241Pro	I	17 y	Focal	Hypotonia	Mild	NA	NA	Normal
#13 (22 y; F)	20	Lys499Asn	II	1.6 y	Focal	Ataxia	Moderate	Hpx	Yes	Removal results of cerebellar astrocytoma
#14 (20 y; M)	5	Lys483Asn	II	18 y	Focal	Hypotonia	Moderate	No	No	Normal
#15 (19 y; F)	15	Leu485Phe	II	14 y	Focal	Tetraparesis, strabismus	Severe/AT	Blinking	Yes	CA
#16 (15 y; F)	10	Trp531Cys	II	9 y	Combined focal and generalized	Tetraparesis, strabismus	Severe	Hpx	Yes	Thin CC
#17 (15 y; F)	8	Leu525Pro	II	11 y	Focal	Tetraparesis, strabismus	Moderate	Hpx	Yes	CA, gliosis
#18 (12 y; F)	2	Gln257Arg	I	10 y	Focal	Ataxia	Severe/AT	No	Yes	Gliosis
#19 (12 y; F)	4	Gln257Arg	I	10 y	Focal	Ataxia	Severe	Blinking	Yes	CA, HS
# 20 (8 y; F)	7	Gln257Arg	I	6 y	Combined focal and generalized	Tetraparesis, strabismus	Severe/AT	No	No	Normal
#21 (8 y; F)	5	Thr599Arg	II	6 y	Combined focal and generalized	Tetraparesis, strabismus	Severe/AT	Hpx	Yes	Gliosis
#22 (4 y; F)	3	Gln257Arg	I	4 y	Generalized	Ataxia, strabismus	Severe	No	Yes	Ventriculomegaly
**Group 3**										
#23 (53 y; F)	7	Ala246Pro	I	-	-	Normal	NA	No	NA	NA
#24 (43 y; F)	10	Thr241Met	I	-	-	Normal	NA	No	NA	NA
#25 (27 y; F)	6	Thr241Arg	I	-	-	Normal	Mild	NA	NA	NA
#26 (24 y; F)	17	Gln709Arg	I	-	-	Normal	Mild	No	Yes	Normal
#27 (19 y; M)	14	Glu501Lys	II	-	-	Normal	Severe	NA	NA	NA
#28 (15 y; M)	6	Leu245Phe	I	-	-	Normal	Mild	NA	NA	NA
#29 (14 y; F)	8	Gly469Glu	II	-	-	Normal	Moderate	No	NA	Thin CC
#30 (12 y; M)	2	Thr470Pro	II	-	-	Normal	Moderate	No	No	Gliosis
#31 (11 y; F)	7	Gln257Arg	I	-	-	Normal	Moderate	NA	NA	Thin CC
#32 (7 y; F)	5	Gln257Arg	I	-	-	Normal	Moderate	No	Yes	Thin CC, cerebellar malrotation
#33 (7 y; F)	5	Trp531Arg	II	-	-	Normal	Moderate	No	Yes	Normal
#34 (3 y; F)	2	Asn581Asp	II	-	-	Normal	Mild	No	NA	NA

y: year; ^†^: patient dead; ID: intellectual disability; PNE: paroxysmal non-epileptic events; SD: sleep disorder; M: male; Hpx: hyperekplexia; ID: intellectual disability; CA: cortical atrophy; HS: hippocampal sclerosis; AT: autistic traits; NA: not available; m: months; F: female; CC: corpus callosum.

## Data Availability

The data that support the findings of this study are available on request from the corresponding author.

## Supplementary Materials

The following are available online at https://www.mdpi.com/article/10.3390/genes12091316/s1, The detailed characterization of the study cohort. Figure S1: Group 1: Interictal EEG in a 12-year-old boy (Pt5, Lys601Gln). Figure S2: Group 2: Interictal EEG in a 9-year-old girl (Pt16, Trp531Cys). Table S1: Genetic and electroclinical characterization of the study cohort. Table S2: Epileptic features of patients with CFCS-causing BRAF mutations reported in the literature.
