# Supplementary material for: Epilepsy and BRAF Mutations: Phenotypes, Natural History and Genotype-Phenotype Correlations
Source: Genes (Basel). 2021 Aug 26;12(9):1316. doi: 10.3390/genes12091316 (PMC8470450; doi:10.3390/genes12091316)
Supplement: Supplementary file 1 [file genes-12-01316-s001.zip › genes-1334004-supplementary.pdf]

Supplemental Data

Supplemental Methods

Figure S1. Group 1: Interictal EEG in a 12-year-old boy (Pt5, Lys601Gln).

Figure S2. Group 2: Interictal EEG in a 9 year-old girl (Pt16, Trp531Cys).

Table S1. Genetic and electroclinical characterization of the study cohort.

Table S2. Epileptic features of patients with CFCS-causing BRAF mutations reported in the literature.

Supplemental Methods

Study Population

Patients belonged to two cohorts: the first one included 23 consecutive patients, who had been referred to the Unit of Pediatric Neurology, Fondazione Policlinico Universitario Agostino Gemelli, Rome, from 1997 to 2018. Nineteen of them had been followed prospectively. The second cohort comprised 11 unselected patients referred to the Unit of Neurology, Ospedale Pediatrico Bambino Gesù, Rome (2000-2018). The study population included 11 males and 23 females, with a mean age of 15.8 years ± 10.6 (range 2 - 53 years). One patient (Pt3) had been treated by left temporal lobectomy for focal cortical dysplasia at 4 years of age and another one (Pt 13) had been treated by lesionectomy for cerebellar astrocytoma at 13 years and at 18 years for recurrence of the lesion. Two patients (Pt7 and Pt9) died at 7 years, during a refractory status epilepticus (RSE), while a single patient (Pt1) died at 23 years from acute respiratory failure.

The mean follow-up period was 9.2 years ± 4.7 (range 2-23 years). Cognitive evaluation was performed by using the Wechsler scales (WISC III, WIPPSI III), Leiter-R and Griffith's Mental Developmental Scales, according to the age of patients and their cooperation (17 out of 31 patients). ID was classified according to the DSM 5 criteria.

All patients shared pathogenic *BRAF* variants (Table 1), which had been documented to occur as *de novo* events and were *bona fide* disease-causing mutations based on the American College of Medical Genetics and Genomics (ACMG) criteria [15]. The relative frequency of each mutation was in line with the data collected in the NSEuroNet database (https://nseuronet.com), a public repository dedicated to RASopathies continuously monitoring the growing literature on the molecular aspects of these disorders.

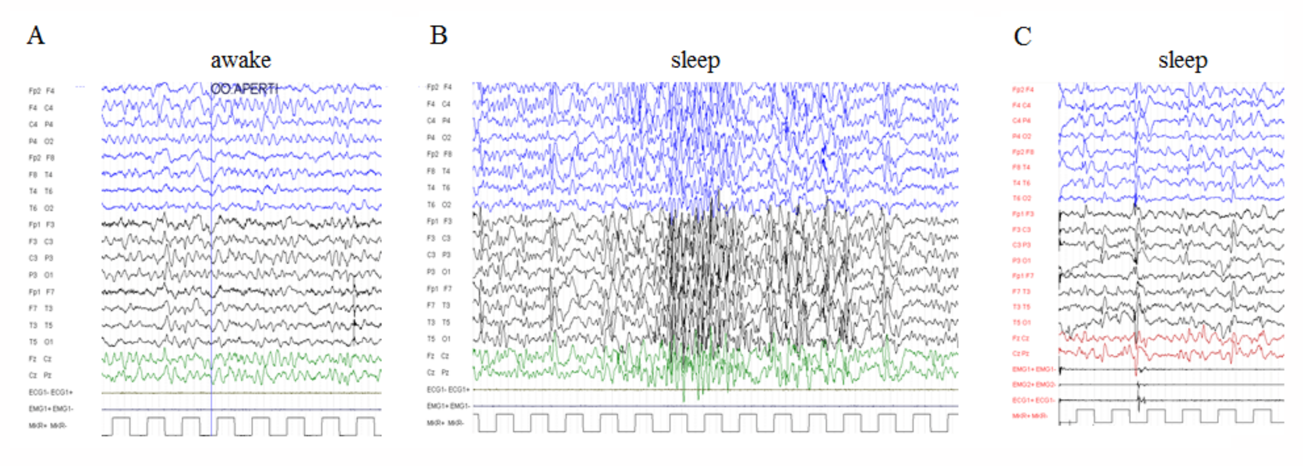

Figure S1. Group 1: Interictal EEG in a 12-year-old boy (Pt5, Lys601Gnl). (**A**) Slow background activity during awake. (**B**) Discharges of irregular, high voltage, diffuse, synchronous and asyncronous SW and polySW during sleep, and discharges of slow waves with superimposed spikes. (**C**) Diffuse discharge of SW sometimes accompanied by isolated myoclonic jerk visible on the right deltoid EMG, during sleep.

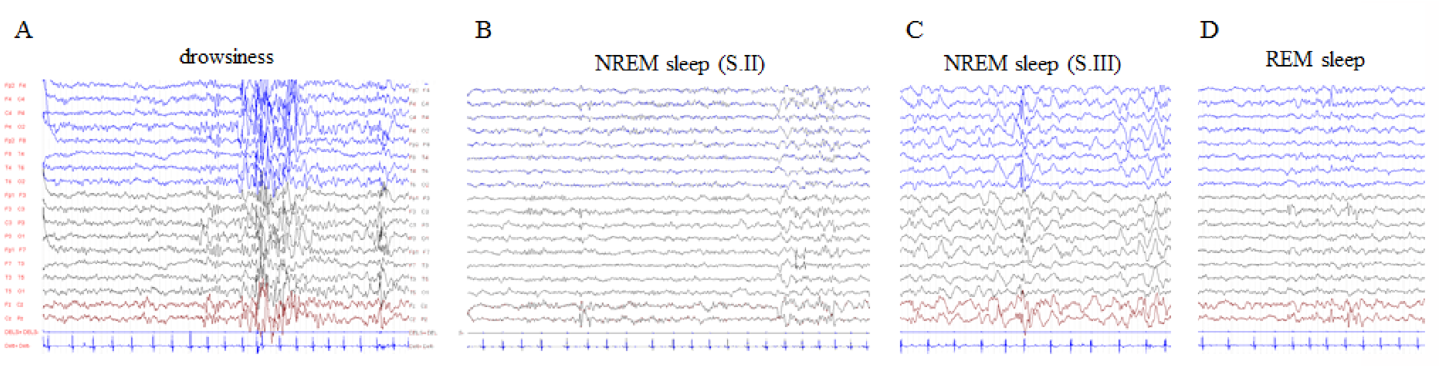

Figure S2. Group 2: Interictal EEG in a 9 year-old girl (Pt16, Trp531Cys). (**A**) Drowsiness: diffuse SW and polySW discharge, predominant on the right regions. (**B**) Asyncronous SW on the both central, left anterior regions and vertex, during stage II of NREM sleep. (**C**) Discharge of SW predominant on the right regions during NREM sleep (Stage III). (**D**) Asyncronous SW on the both central, left anterior regions and vertex during REM Sleep.

Table S1. Genetic and electroclinical characterization of the study cohort.

GROUP (age;sex)	F-UP (y)	BRAF amino acid substitutionand mutation class	Age and sz. type at onset	Duration of sz. free period after onset	Seizure type	SE (frequency/ type)	Epilepsy type	EEG (background activity at outcome)	EEG (paroxysmal activity at outcome)	sz. frequency at outcome	AEDs
**Group 1**											
#1 (23y; M†)	23	Lys601Gnl II	2m focal onset	6y	I) Focal onset: - motor/hyperkinetic - focal to bilateral tonic- clonic. II) Generalized onset: - myoclonic	Recurrent/ convulsive and focal motor	Combined focal and generalized	Slow, asymmetric	Multifocal and diffuse S, SW and polySW; bursts of focal fast activity	Daily	PB, VPA, CBZ, BDZ, LTG, LEV, TPM, ZNS, PHT, RUF
# 2 (17y; M)	15	Asp638Glu III	2y focal onset	no	I) Focal onset: - motor - focal to bilateral tonic- clonic. II) Generalized onset: - epileptic spasms	Recurrent/ focal motor	Combined focal and generalized	Slow	Multifocal and diffuse S and SW in awake; alternating pattern in sleep; bursts of focal fast activity	Daily	PB, CBZ, GVG, BDZ
# 3 (15y; M)	15	Asp638Glu III	5m epileptic spasms	8m	I) Focal onset: - motor/hyperkinetic - focal to bilateral tonic- clonic. II) Generalized onset: - epileptic spasms - tonic	Recurrent/ convulsive and focal motor	Combined focal and generalized	Slow	Multifocal and diffuse S and SW in awake; alternating pattern in sleep	Daily	CBZ, TPM, LEV, PHT, BDZ
#4 (14y; F)	12	Phe595Leu II	1.5y focal onset	5y	Focal onset: - motor/hyperkinetic - focal to bilateral tonic- clonic	Recurrent/ focal motor, RSE	Focal	Slow	Focal S and SW	Weekly	CBZ, LEV, PB, TPM, PRP, BDZ
#5 (13y; M)	11	Lys601Gnl II	1.5y focal onset	1.5y	I) Focal onset: - motor/hyperkinetic - epileptic spasms. II) Generalized onset: - myoclonic - epileptic spasms	Recurrent/ focal motor	Combined focal and generalized	Slow	Multifocal S, SW and polySW; diffuse discharges of slow W with superimposed S; bursts of focal fast activity	Weekly/ monthly	PB, CBZ, BDZ
#6 (12y; F)	7	Asp638Glu III	1day focal onset	1.5y	I) Focal onset : - motor - focal to bilateral tonic- clonic. II) Generalized onset:	Recurrent/ focal motor, RSE	Combined focal and generalized	Slow, asymmetric	Focal and diffuse S, SW	Weekly / monthly	VPA, BDZ, LEV, LCM, PHT

					- myoclonic						
#7 (7y; F †)	6	Phe595Leu II	7m focal onset	3y	I) Focal onset: - motor/hyperkinetic - focal to bilateral tonic- clonic. II) Generalized onset: -myoclonic	Recurrent/ focal motor, RSE	Combined focal and generalized	Slow, asymmetric	Multifocal S, SW and polySW; diffuse discharges of slow W with superimposed S	Recurrent RSE followed by exitus	PB, ACTH, CBZ, BDZ, LCS, LEV, RUF, BDZ, VNS.
#8 (6y; F)	6	Val487Gly I	8day focal onset	7y	I) Focal onset: - motor/hyperkinetic - focal to bilateral tonic- clonic	Recurrent/ focal motor	Focal	Slow	Focal S and SW; diffuse discharges of slow W with superimposed S; bursts of focal fast activity	Weekly/ monthly	VPA, BDZ, LCS
#9 (5y; M †)	NA	Asp565Glu I	4y unknown onset	no	I) Focal onset: - motor/hyperkinetic - focal to bilateral tonic-clonic. II) Generalized onset: -myoclonic	Recurrent/ focal motor, RSE	Combined focal and generalized	NA	NA	Recurrent RSE followed by exitus	PB, VPA, LEV, PHT
#10 (3y;M)	3	Pro468Ser II	1m epileptic spasms	5y	Focal onset: - motor - focal to bilateral tonic- clonic - epileptic spasms - myoclonic	Recurrent/ - febrile and afebrile focal motor; - myoclonic	Focal	Slow	Multifocal and diffuse S and SW in awake; alternating pattern in sleep; bursts of focal fast activity	Daily	VPA, TPM, PB, BDZ
**Group 2**											
#11 (27y;F)	25	Trp531Cys II	2y focal onset	No	Focal onset: - focal to bilateral tonic- clonic.	No	Focal	Normal	Focal S and sharp W	Seizure free	LEV
#12 (23y;M)	10	Thr241Pro I	17y focal onset	No	Focal onset: - focal to bilateral tonic-clonic.	No	Focal	Normal	Focal S and sharp W	Seizure free	LTG
#13 (22y;F)	20	Lys499Asn II	1,6y focal onset	No	Focal onset: - focal to bilateral tonic- clonic	No	Focal	Normal	Giant spindles; fast activity on anterior regions; focal S	Seizure free	PB
#14 (20y;M)	5	Lys483Asn II	18y focal onset	No	Focal onset : - focal to bilateral tonic-clonic	No	Focal	Normal	No	Seizure free	No AED
#15 (19y;F)	15	Leu485Phe II	14y focal onset	No	Focal onset: - focal to bilateral tonic-clonic	No	Focal	Normal	Giant spindles; focal S	Seizure free	VPA
#16 (15y;F)	10	Trp531Cys II	9y myoclonic	No	I) Focal onset: - focal to bilateral tonic-clonic. II) Generalized onset: - myoclonic	No	Combined focal and generalized	Normal	Focal and diffuse S and SW	Seizure free	VPA

#17 (15y;F)	8	Leu525Pro II	11y focal onset	No	Focal onset : - focal to bilateral tonic- clonic	No	Focal	Normal	Rare multifocal S	Seizure free	CBZ
#18 (12y;F)	2	Gln257Arg I	10y focal onset	No	Focal onset: - motor	No	Focal	Normal	Fast activity; focal S and sharp W	Seizure free	OXC
#19 (12y; F)	4	Gln257Arg I	10y focal onset	No	Focal onset: - motor - focal to bilateral tonic- clonic	Isolated preAED/ focal motor	Focal	Normal	Focal S and sharp W	Seizure free	VPA, BDZ
# 20 (8y; F)	7	Gln257Arg I	6y focal onset	No	I) Focal onset: - motor. II) Generalized onset: -myoclonic	Isolated, preAED/ focal motor	Combined focal and generalized	Normal	Focal S and sharp W	Seizure free	CBZ
#21 (8y;F)	5	Thr599Arg II	6y focal onset	No	I) Focal onset: - motor. II) Generalized onset: - myoclonic	No	Combined focal and generalized	Normal	Focal sharp W	Seizure free	VPA
#22 (4y;F)	3	Gln257Arg I	4y myoclonic	No	Generalized onset: - myoclonic	No	Generalized	Normal	Giant spindles; theta rhythmic activity in sleep; diffuse S and SW	Seizure free	No AED

y: years; Mut: mutation; sz: seizure; SE: status epilepticus; AEDs: antiepileptic drugs; M: male; m: months; S: spike; SW: spike wave; polySW: poly spike wave; PB phenobarbital; VPA: valproic acid; CBZ: carbamazepine; BDZ: benzodiazepine; LTG: lamotrigine; LEV: leveracetam; TPM: topiramate; ZNS: zonisamide; PHT: phenytoine; RUF: rufinamide; GVG: vigabatrin; OXC: oxcarbazepine; F: female; RSE: refractory status epilepticus; PRP: perampanel; LCM: lacosamide; W: wave; ACTH: synacthen; VNS: vagus nervus stimulator; NA: not available.

†: patient deceased.

Table S2. Epileptic features of patients with CFCS-causing *BRAF* mutations reported in the literature.

Patient (age; sex)	Onset	Group	Epilepsy features	Mutation	Mutation class	Reference
#L1 (7y; M)	2w	1	Polymorphic seizures (two or more seizure types), drug resistant	L485F	II	Yoon et al 2007 [1]
#L2 (12.1y; F)	11y	1	Polymorphic seizures (two or more seizure types), drug resistant	F468S	II	Yoon et al 2007 [1]
#L4 (7.6y; F)	6m	1	Polymorphic seizures (two or more seizure types), drug resistant	F595L	II	Yoon et al 2007 [1]

#L5 (2.2y; M)	4m	1	Polymorphic seizures (two or more seizure types), drug resistant	L485S	II	Yoon et al 2007 [1]
#L6 (14y; F)	1y 6m	1	Polymorphic seizures (two or more seizure types), drug resistant	D638E	III	Yoon et al 2007 [1]
#L7 (20.5y; F)	7m	1	Polymorphic seizures (two or more seizure types), drug resistant	K499N	II	Yoon et al 2007 [1]
#L8 (15y; F)	NA	1	Epileptic abnormalities on EEG, seizures controlled with multidrug therapy	F468S	II	Demir et al 2010 [8]
#L9 (4m; F)	2m	1	Epileptic encephalopathy with infantile spasms, drug resistant	L485S	II	Aizaki et al 2011 [6]
#L10 (1y; F)	1d	1	Polymorphic seizures (two or more seizure types), drug resistant	L485S	II	Adachi et al 2012 [2]
#L11 (6y; F)	4y 4m	1	Recurrent epileptic status, drug resistant, psychomotor regression	Q257R	I	Wakusawa et al 2014 [3]
#L12 (1y; F)	4m	1	Epileptic encephalopathy with infantile spasms, drug resistant	P595L	II	Hatory et al 2016 [9]
#L13 (2.6y; M)	4m	1	Polymorphic seizures (two or more seizure types), early onset, drug resistant	Leu485del	II	Suzuki-Muromoto et al 2019 [5]
#L14 (10.10y; F)	2y6m	2	Drug responsive epilepsy, treated with only one drug	Q257R	I	Yoon et al 2007 [1]
#L15 (8y; F)	3y	2	Drug responsive epilepsy, treated with only one drug	Q257R	I	Yoon et al 2007 [1]
#L16 (5.6y; F)	3y	2	Drug responsive epilepsy, treated with only one drug	T599R	II	Yoon et al 2007 [1]
#L17 (7.3y; M)	5y	2	Drug responsive epilepsy, treated with only one drug	G534R	II	Yoon et al 2007 [1]
#L18 (4y; M)	4y	2	Febrile seizures, normal EEG	Q257R	I	Papadopoulu 2011 [7] cited by Yoon 2007 [1]
#L19 (7y; F)	-	3	No seizures	E275K	I	Sarkozy et al 2009 [4]
#L20 (3m; M)	-	3	No seizures	L245F	I	Sarkozy et al 2009 [4]
#L21 (1.8y; F)	-	3	No seizures	Q257R	I	Sarkozy et al 2009 [4]
#L22 (8 m; F)	-	3	No seizures	G469E	II	Sarkozy et al 2009 [4]
#L23 (7y; F)	-	3	No seizures	L485F	II	Sarkozy et al 2009 [4]
#L24 (5.3y; M)	-	3	No seizures	E501K	II	Sarkozy et al 2009 [4]
#L25 (2.3y; F)	-	3	No seizures	L525P	II	Sarkozy et al 2009 [4]

#L26 (12.3y; F)	-	3	No seizures	Q709R	II	Sarkozy et al 2009 [4]
#L27 (2y; M)	-	3	No seizures	T244P	I	Gripp et al 2007 [10]
#L28 (1y; F)	-	3	No seizures	Q257R	I	Gripp et al 2007 [10]
#L29 (1y; M)	-	3	No seizures	Q257R	I	Gripp et al 2007 [10]
#L30 (3y; M)	-	3	No seizures	L525P	II	Gripp et al 2007 [10]
#L31 (2y; M)	-	3	No seizures	D565E	II	Gripp et al 2007 [10]
#L32 (6y; F)	-	3	No seizures, normal EEG	E501K	II	Demir et al 2010 [8]
#L33 (3y; F)	-	3	No seizures	D638E	III	Demir et al 2010 [8]

M: male; F: female; w: week; y: year; m: months.

References:

1. Yoon G, Blaser J, Rauen KA. Neurological complications of cardio-facio-cutaneous syndrome, Development Medicine & Child Neurology. **2007;** 49:894-9.
2. Adachi M, Abe Y, AokiY, Matsubara Y. Epilepsy in RAS/MAPK syndrome: two cases of cardio-facio-cutaneous syndrome with epileptic encephalopathy and a literature review. Seizures. **2012**; 21:55-60.
3. Wakusawa K, Kobayashi S, Abe Y, Tanaka S, Endo W, Inui T, et al. A girl with Cardio-facio-cutaneous syndrome complicated with status epilepticus and acute encephalopathy. Brain Dev. **2014;** 36:61-3.
4. 22 Sarkozy A, Carta C, Moretti S, Zampino G, Digilio MC, Pantaleoni F et al. Germline BRAF mutations in Noonan, LEOPARD, and cardiofaciocutaneous syndromes: molecular diversity and associated phenotypic spectrum. Hum Mutat. **2009**; 30:695-702.
5. Suzuki-Muromoto, S.; Miyabayashi, T.; Nagai, K.; Yamamura-Suzuki, S.; Anzai, M.; Takezawa, Y.; Sato, R.; Okubo, Y.; Endo, W.; Inui, T. Leucine-485 deletion variant of BRAF may exhibit the severe end of the clinical spectrum of CFC syndrome. *J. Hum. Genet.* **2019**, *64*, 499–504.
6. Aizaki, K.; Sugai, K.; Saito, Y.; Nakagawa, E.; Sasaki, M.; Aoki, Y.; Matsubara, Y. Cardio-facio-cutaneous syndrome with spasms and delayed myelination. *Brain Dev.* **2011**, *33*, 166–169.
7. Papadopoulou, E.; Sifakis, S.; Sol-Church, K.; Klein-Zighelboim, E.; Stabley, D.L.; Raissaki, M.; Gripp, K.W.; Kalmanti, M. CNS imaging is a key diagnostic tool in the evaluation of patients with CFC syndrome: Two cases and literature review. *Am. J. Med. Genet. A* **2011**, *155*, 605–611.
8. Demir E, Mancano G, Pomponi MG, Ozcelik A, Gucuyener K, Neri G.Cardio-facio-cutaneous syndrome: phenotypic variability and differential diagnosis in 3 cases with de novo BRAF mutations. Neuropediatrics. **2010**; 41:127-31.
9. Hatori T, Sugiyama Y, Yamashita S, Hirakubo Y, Nonaka K, Ichihashi K. Vigabatrin Therapy for Infantile Spasms in a Case of Cardiofaciocutaneous Syndrome with Cardiac Hypertrophy Developing during Adrenocorticotropic Hormone Treatment. J Nippon Med Sch. **2016**; 83: 167-71.

10. Gripp KW, Lin AE, Nicholson L, Allen W, Cramer A, Jones K et al. Further delineation of the phenotype resulting from BRAF or MEK1 germline mutations helps differentiate cardio-facio- cutaneous syndrome from Costello syndrome. Am J Med Genet A. **2007**; 143A:1472-80.
